# Signals for Hepatic Figrogenesis in Pediatric Cholestatic Liver Disease: Review and Hypothesis

**DOI:** 10.1186/1476-5926-2-S1-S5

**Published:** 2004-01-14

**Authors:** Grant A Ramm, Anita C Hoskins, Sonia A Greco, Tamara N Pereira, Peter J Lewindon

**Affiliations:** 1 The Hepatic Fibrosis Group, The Queensland Institute of Medical Research and The University of Queensland, Brisbane, Queensland 4029, Australia

## Neonatal Cholestatic Liver Diseases

Cholestatic liver disease in children occurs as a result of either an alteration in hepatocyte bile formation or disruption of bile flow out of the hepatocyte through intrahepatic bile ductules or extrahepatic bile ducts [1]. Liver disease usually appears within the first few weeks following birth. A large number of disorders exhibit cholestatic jaundice in neonatal life including (a) numerous cholangiopathies, such as extrahepatic biliary atresia, cystic fibrosis (CF), choledochal cyst, alpha_1_-Antitrypsin deficiency and Alagille's syndrome, (b) several abnormalities of the gall bladder, such as cholelithiasis and cholecystitis, and (c) bile acid transport disorders. The most commonly occurring form of neonatal cholestasis is biliary atresia, representing a relative frequency of approximately 30% [1]. In order to administer effective therapeutic intervention early diagnosis is critical. This can prove difficult as a number of phenotypic manifestations of the many different forms of neonatal cholestasis are similar and may even overlap.

## Role of Hepatic Stellate Cells

If left untreated cholestatic liver injury results in the development of hepatic fibrosis and ultimately may progress to cirrhosis. The precise pathophysiological mechanisms responsible for hepatic fibrogenesis in pediatric cholestatic liver disease are unknown. Hepatic stellate cells have been shown to be responsible for increased production of fibrillar collagens in a number of adult liver diseases and in experimental models of liver injury. Recent studies by our group have identified stellate cells as the principal source of type I collagen in neonatal cholestatic liver diseases such as biliary atresia [2] and in the focal biliary cirrhosis associated with CF [3]. In these studies, large numbers of myofibroblast-like cells were demonstrated in the extracellular matrix surrounding expanded bile ducts and within fibrosis septa bridging between portal tracts (2). Activated hepatic stellate cells, demonstrated by the expression of –-smooth muscle actin and their stellate morphology, were particularly evident at the interface between scar and normal tissue [2,3]. This growing margin of scar tissue formation appeared to be the site of maximal stellate cell activation and type I collagen mRNA expression, although some evidence of collagen gene expression was also seen in myofibroblast-like cells within established fibrous septa. This clearly suggests a role for hepatic stellate cells in the deposition of fibrillar collagens as the scar expands with continued cholestatic liver injury. Whether the myofibroblast-like cells which surround expanded bile ducts within the fibrotic septa are derived from activated hepatic stellate cells or portal fibroblasts remains unclear.

## Mediators of Fibrogenesis

**Figure 1:**
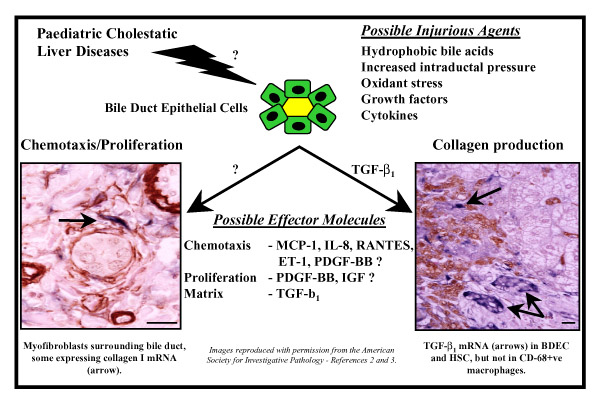
Paediatric Cholestasis – Potential Initiating and Perpetuating Factors: TGF-beta_1_, Transforming Growth Factor-beta_1_; MCP-1, Monocyte Chemotaxis Protein-1; IL-8, Interleukin-1; ET-1, Endothelin-1; PDGF-BB, Platelet-Derived Growth Factor-BB; IGF, Insulin-like Growth Factor. Bar: 50 –m.

The precise mechanisms responsible for the activation of hepatic stellate cells and the subsequent development of hepatic fibrosis in pediatric cholestatic liver disease are not known. Bile duct hyperplasia appears to be an early event in the pathology of both biliary atresia and CF liver disease. It has been suggested that the biliary hyperplasia seen in bile duct-ligated rats may be due to either increased intraductal pressure [4] or circulating cholangiotrophic factors [5]. Hepatic cell injury is suggested as a potential cause of fibrogenesis in cholestatic liver disease. This may be due to an increase in the levels of glycine-conjugated hydrophobic bile acids which occurs in response to biliary obstruction and decreased hydration of bile [6]. It has also been suggested that cholestatic hepatotoxicity may be induced via the depletion of hepatic antioxidants, such as vitamin E or glutathione [7]. Thus, hydrophobic bile acid-induced oxidant stress may play a major role in the viability and function of Kupffer cells, hepatocytes, bile duct cells and hepatic stellate cells, all of which are capable of producing cytokines and growth factors which may drive inflammation and fibrogenesis (Figure 1).

## Transforming Growth Factor-beta1 and Matrix Production

**Figure 2:**
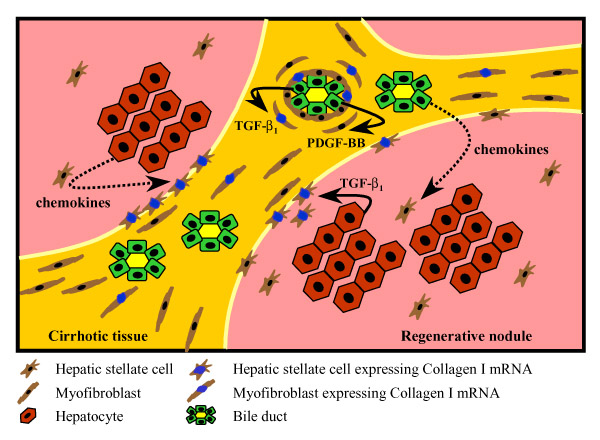
Paediatric Cholestasis – Chemotaxis, Mitogenesis and Matrix Production.

While the precise mechanisms responsible for initiating fibrogenesis in pediatric cholestasis are unclear, it is known that a number of cytokines contribute to the perpetuation of this process. We have recently demonstrated that the expression of the profibrogenic cytokine, transforming growth factor-beta 1 (TGF-beta_1_), is significantly increased in both biliary atresia [2] and CF liver disease [3], and is predominantly expressed by bile duct epithelial cells and also by hepatocytes at the growing margin of the scar tissue [2,3]. We hypothesize that this TGF-beta_1 _is responsible for the transactivation of the procollagen alpha_1_(I) gene in both myofibroblast-like cells and activated hepatic stellate cells in these same areas of scar tissue formation (Figures 1 and 2). The expression of TGF-beta_1 _at the scar interface, where maximal fibrogenic activity occurs in both biliary atresia and CF liver disease, suggests both a spatial and temporal association in the generation of scar tissue in these cholestatic liver diseases. Indeed, our studies have shown that TGF-beta_1 _protein expression in bile duct epithelial cells is significantly correlated with the percentage of portal tracts involved in the histological abnormalities associated with focal biliary cirrhosis in CF liver disease and that TGF-beta_1 _mRNA expression correlates with disease progression [3].

## Role of Mitogens and Chemokines

Another key regulator of fibrogenesis is the mitogenic cytokine, platelet-derived growth factor-BB (PDGF-BB). The observation of abundant myofibroblast-like cells within the peri-biliary and scar interface regions of the acinus in both biliary atresia and CF liver disease suggests either local activation and proliferation of portal fibroblasts or recruitment of stellate cells. Therefore, we hypothesize that bile duct epithelial cells and hepatocytes at the scar interface produce mitogens and chemokines in response to biliary obstruction (Figures 1 and 2). There is recent evidence to support a role for PDGF-BB in cholestatic liver injury. In a study using bile duct-ligated rats, Kinnman and colleagues demonstrated that bile duct cells express PDGF-BB [8]. Using an *in vitro *culture system they showed that hepatic stellate cells are recruited to bile duct segments by PDGF-BB [8]. While this is an important observation, the role of PDGF-BB in human neonatal cholestasis has not been evaluated. Hepatic stellate cells also respond to numerous other chemokines *in vitro*, such as monocyte chemotaxis protein-1 (MCP-1), interleukin-8, RANTES, endothelin-1 [9] (Figure 1). It is possible that these chemokines may also play a role in stellate cell recruitment in cholestatic liver disease (Figure 2). Marra and colleagues have reported that MCP-1 causes stellate cell recruitment *in vitro *[10] and MCP-1 expression is increased in cirrhotic adult liver [11]. We propose that MCP-1 may play a major role in the development of pediatric cholestatic liver injury. In preliminary studies, we have reported marked hepatic expression of MCP-1 in biliary atresia and CF liver disease, although the role of MCP-1 in stellate cell recruitment *in vivo *remains to be determined [12].

## Conclusions

While the precise pathways involved in the initiation of hepatic fibrogenesis in neonatal cholestasis remain elusive, a number of cytokines and growth factors have been identified which contribute to the progression of injury through matrix production and recruitment of inflammatory cells. With this knowledge it may be possible to develop novel approaches for therapeutic intervention which could assist in the suppression of fibrogenesis and may potentially lead to the reduced requirement for liver transplantation.
